# Prognostic Significance of the Fission1/Parkin Ratio for Sepsis: A Prospective Cohort Study

**DOI:** 10.3389/fmed.2021.642749

**Published:** 2021-05-13

**Authors:** Wei Huang, Xiaoting Wang, Hongmin Zhang, Guangjian Wang, Dawei Liu

**Affiliations:** Department of Critical Care Medicine, Peking Union Medical College Hospital, Peking Union Medical College, Chinese Academy of Medical Sciences, Beijing, China

**Keywords:** mitochondrial homeostasis, fission1/parkin ratio, sepsis, biomarker, prognosis

## Abstract

**Introduction:** Fission1 (Fis1) and parkin are key proteins related to mitochondrial fission and mitophagy, respectively. This study aimed to assess the prognostic value of the Fis1/parkin ratio as a biomarker in patients with sepsis.

**Methods:** Consecutive patients with sepsis (*n* = 133) or simple infection (*n* = 24) were enrolled within 24 h of arrival at the intensive care unit (ICU). Serum levels of Fis1, parkin, mitofusin2 (Mfn2), and peroxisome proliferator-activated receptor γ coactivator 1α (PGC-1α) were measured by enzyme-linked immunosorbent assay (ELISA) upon ICU admission. Clinical parameters and standard laboratory test data were also collected. All patients received follow-up for at least 28 days.

**Results:** Patients with sepsis presented with significantly decreased serum levels of parkin, Mfn2, and PGC-1α, but an increased serum Fis1 level and Fis1/parkin, Fis1/Mfn2, and Fis1/PGC-1α ratios at ICU admission. Relative to patients with simple infections, the ratios were remarkably elevated in septic patients—particularly septic shock patients. The area under the receiver operating characteristic (ROC) curve of the Fis1/parkin ratio was greater than that of Fis1, parkin, Mfn2, and PGC-1α levels as well as that of the Fis1/Mfn2 and Fis1/PGC-1α ratios for prediction of 28-day mortality due to sepsis. All of the ratios were significantly higher in non-survivors than survivors at the 28-day follow-up examination. Fis1/parkin ratio was found to be an independent predictor of 28-day mortality in patients with sepsis.

**Conclusions:** The Fis1/parkin ratio is valuable for risk stratification in patients with sepsis and is associated with poor clinical outcomes for sepsis in the ICU.

## Introduction

Sepsis is life-threatening organ dysfunction caused by a dysregulated host response to infection (1). Each year there are an estimated 48.9 million incident cases of sepsis worldwide and 11.0 million sepsis-related deaths, representing 19.7% of all global deaths (2). However, effective management of sepsis and resource allocation remain a challenge due to the inability to accurately diagnose the severity and risk of sepsis. Therefore, prognostic biomarkers are needed for early identification of patients at high risk of sepsis. Such patients could be transferred to the ICU and receive optimized hospital resources and therapies.

Among the complex mechanisms of sepsis and its heterogeneous nature, defective mitochondrial quality control (MQC) plays an important role in the severity of sepsis and sepsis-induced multiple organ dysfunction syndrome (MODS) (3–6). The MQC system aims to maintain mitochondrial homeostasis, allowing the mitochondrial network to segregate, recognize, and eliminate damaged mitochondria and to generate new mitochondria. MQC processes include mitochondrial biogenesis, mitochondrial dynamics (mitochondrial fission and fusion), and mitophagy (7, 8). Previous studies have demonstrated that sepsis is ameliorated by the recovery of mitochondria homeostasis (4, 9, 10). Therefore, indicators related to mitochondrial homeostasis may be useful for risk stratification and prognostic evaluation of patients with sepsis.

Fission 1 (Fis1), parkin, mitofusin2 (Mfn2), and peroxisome proliferator-activated receptor γ coactivator 1α (PGC-1α) are four key proteins involved in mitochondrial fission, mitophagy, mitochondrial fusion, and mitochondrial biogenesis, respectively (10). It has been shown that recovery of mitophagy, mitochondrial fusion, and biogenesis can partly reverse organ failure under septic conditions, whereas worsening of sepsis is accompanied by activation of mitochondrial fission (3, 11–14). In other words, elevations of parkin, Mfn2, and PGC-1α appear to protect against organ dysfunction in animal models of sepsis, whereas Fis1 is associated with sepsis severity and multiple organ dysfunction.

The peripheral blood samples are an attractive tissue for biomarker discovery as they are easily obtained and analyzed. It has been proved that the expression of Fis1, Mfn2, parkin and PGC-1α in peripheral blood mononuclear cells (PBMCs) could give us some information about the mitochondrial quality control status (15–19), and considering the immune cell death during sepsis (20, 21), MQC-related proteins in the immune cells could continuously release into circulation, making the detection of these proteins in serum possible.

Simultaneous measurement of multiple biomarkers may be useful for overcoming the limitations of using a single biomarker. Assessment of multiple biomarkers associated with different sepsis-related pathways may be particularly useful. Thus, in the present study, we use the Fis1/parkin, Fis1/Mfn2, and Fis1/ PGC-1α ratios to reflect the severity of mitochondrial homeostasis disbalance. This study aimed to investigate the feasibility of using the Fis1/parkin, Fis1/Mfn2, and Fis1/ PGC-1α ratios to predict the prognosis of septic patients and to identify what ratio provided the best performance.

## Materials and Methods

### Patients

This prospective study was carried out at Peking Union Medical College Hospital (PUMCH) between June 2019 and August 2020. The study was approved by the PUMCH institutional review board (approval number JS-2421) and informed consent was obtained from all enrolled patients or their relatives. Patient records were anonymized and deidentified before analysis.

The inclusion criteria were: (1) age ≥18 years and (2) diagnosis of sepsis [according to The Third International Consensus Definitions for Sepsis and Septic Shock (Sepsis-3) (1)]. The exclusion criteria were: (1) age <18 years, (2) massive bleeding or pulmonary embolism, (3) heart attack or acute exacerbation of previous heart disease in the previous week, (4) heart surgery in the previous week, and (5) lack of informed consent by the patient or their relatives.

A total of 133 septic patients, who were followed for 28 days or until death, were enrolled in this study. Septic patients were divided into non-shock and shock subgroups. The criteria for inclusion in the shock subgroup were: a clinical construct of sepsis with persisting hypotension requiring vasopressors to maintain MAP ≥ 65 mmHg and a serum lactate level >2 mmol/L (18 mg/dL) despite adequate volume resuscitation on the day of ICU admission (1). Septic patients who did not meet these criteria were assigned to the septic non-shock group. Additionally, 24 patients with simple infection, but who did not meet the criteria for sepsis, admitted to the intensive care unit (ICU) served as a control group.

Baseline clinical and laboratory characteristics were obtained from medical records and routine ICU tests, including patient age, sex, hemodynamic parameters, blood chemistry, arterial blood gas analysis, Acute Physiology and Chronic Health Evaluation (APACHE) II score, and SOFA score.

### Sample Collection and Measurement

Peripheral blood samples were collected within 24 h of ICU admission and centrifuged immediately. Serum was withdrawn and stored at −80°C until assessment by enzyme-linked immunosorbent assay (ELISA). The serum levels of Fis1, parkin, Mfn2, and PGC-1α were determined using commercially available ELISA kits following the instructions of the manufacturer (Fis1: Abbexa abx151559 Cambridge, UK; parkin: Abcam ab212159 Shanghai, China; Mfn2: Abebio AE33636HU Wuhan, China; PGC-1α: Cusabio CSB-E11761h Wuhan, China). According to the manufacturers' specifications, the ELISA assays were specific for native proteins, with no significant cross-reactivity with known analogs. Each well of the ELISA plate was loaded with 100 μL undiluted blood sample, and we did not measure the protein concentration before loading the plate.

### Statistical Analysis

Results are presented as the mean and standard deviation or median and range (interquartile range). Group differences for continuous variables with a non-normal distribution were tested using the Kolmogorov-Smirnov test and Mann–Whitney *U* test. Categorical variables are reported as proportions and compared using the χ^2^ or Fisher's exact tests. Logistic regression analysis was used to determine independent predictors of 28-day mortality. The receiver operating characteristic (ROC) curve was used to assess the accuracy of the variables for prediction of 28-day mortality. The Kaplan–Meier method was used to analyze the survival data. Comparisons between groups were performed using the log-rank test. *P* < 0.05 was considered to be statistically significant. All statistical analyses were performed with SPSS 22.0 software (IBM Inc.).

## Results

### Patient Characteristics

**Figure 1:**
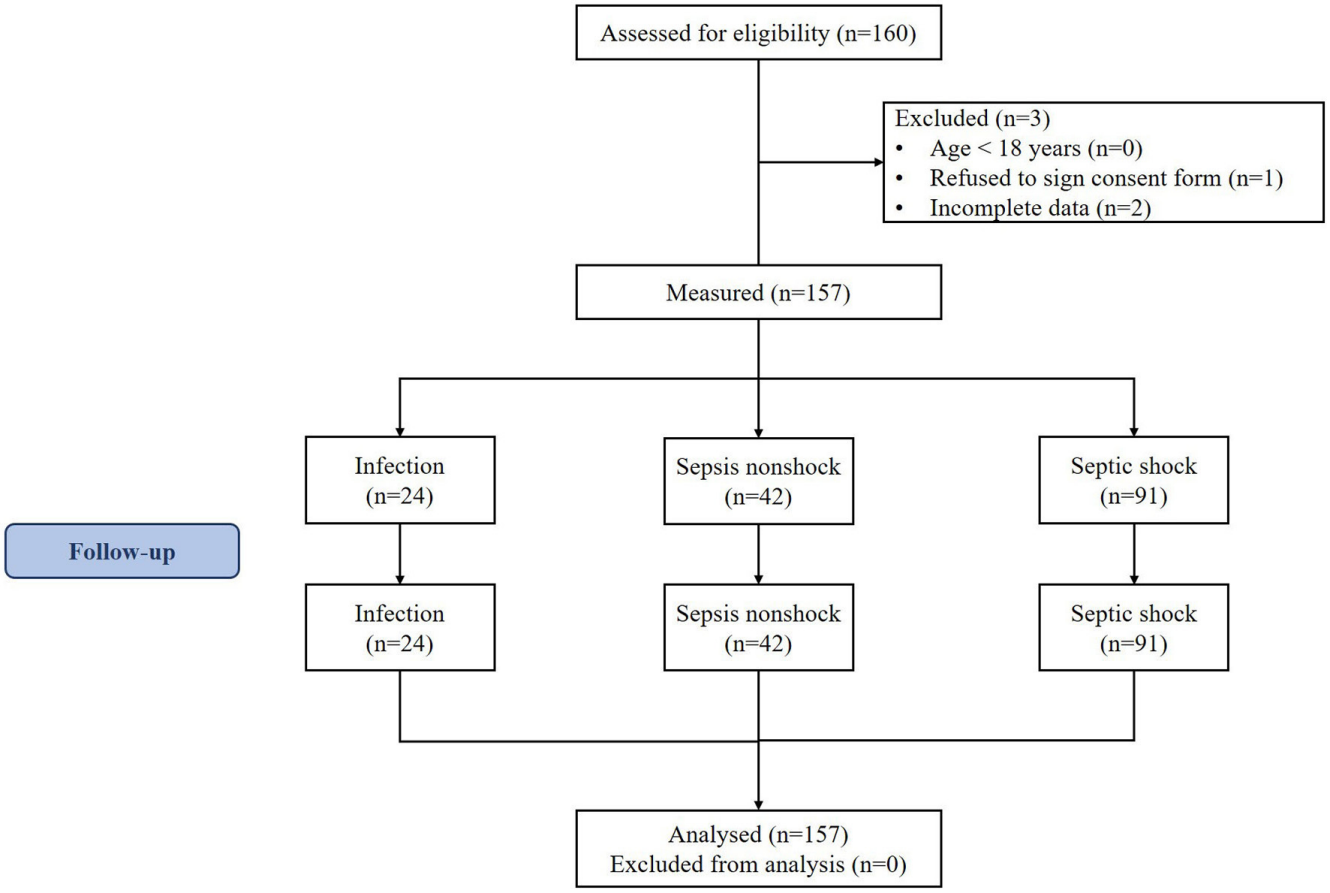
CONSORT flow diagram of the study.

Patient recruitment for this study is shown in Figure 1. A total of 133 septic patients and 24 infected patients (controls) were enrolled in the study.

There were no significant differences in age, central venous blood oxygen saturation (ScvO2), or arteriovenous carbon dioxide partial pressure difference (Pv-aCO2) between the two groups (infection and sepsis). APACHE II and SOFA scores differed between groups, with the sepsis group scoring significantly higher (*p* < 0.001). The basic characteristics all enrolled patients are listed in Table 1.

**Table 1:** Characteristics of the study population.

**Characteristics**	**Infection**	**Sepsis**	***P-*value**
Number of subjects	24	133	
**Demographic data**			
Age (yr)	62 (46–68)	65 (56–72)	0.092
Male, *n* (%)	21 (87.5%)	87 (65.4%)	0.032
**Physiological data**			
MAP (mmHg)	94.87 ± 14.18	85.73 ± 13.84	<0.001
HR (beats/min)	87 (77–100)	100 (86–114)	<0.001
PaO2/FiO2 ratio	356 (281–482)	278 (194–379)	<0.001
Lac (mmol/L)	1.0 (0.7–1.4)	1.6 (1.1–2.4)	0.002
Pv-aCO2 (mmHg)	4.6 (4.4–5.0)	3.7 (2.4–6.3)	0.368
ScvO2 (%)	69.2 (64.0–73.8)	74 (68.4–78.9)	0.252
SOFA score	0.5 (0–1)	12 (9–14)	<0.001
APACHE II score	13.5 (8–17)	20 (16–26)	<0.001
28-day mortality, *n*(%)	0 (0%)	18 (13.5%)	<0.001

*Date was presented by mean ± standard deviation, n (%) or median (interquartile range)*.

*HR, heart rate; MAP, mean arterial blood pressure; Pv-aCO2, arteriovenous carbon dioxide partial pressure difference; ScvO2, central venous blood oxygen saturation; SOFA, sequential organ failure assessment; APACHE II, Acute Physiology and Chronic Health Evaluation II score*.

### Group Differences in Serum Levels and Ratios

**Figure 2:**
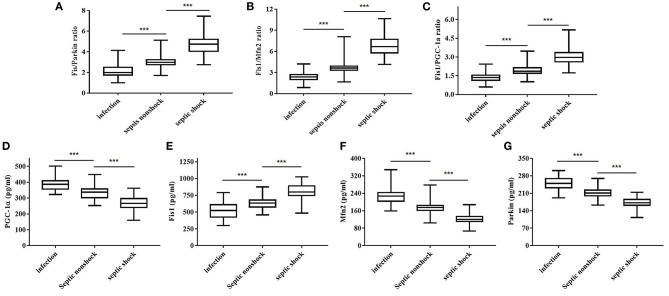
Fis1/parkin ratio **(A)**, Fis1/Mfn2 ratio **(B)**, Fis1/PGC-1α ratio **(C)** and serum PGC-1α **(D)**, Fis1 **(E)**, Mfn2 **(F)**, parkin **(G)** levels at ICU admission in the patient subgroups.

**Figure 3:**
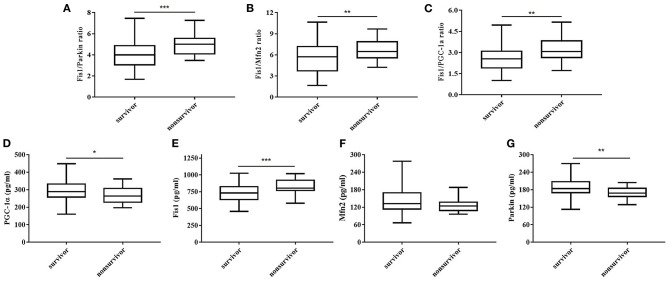
Fis1/parkin ratio **(A)**, Fis1/Mfn2 ratio **(B)**, Fis1/PGC-1α ratio **(C)** and serum PGC-1α **(D)**, Fis1 **(E)**, Mfn2 **(F)**, parkin **(G)** levels at ICU admission in survivor and non-survivor groups of patients with sepsis at 28-day follow-up.

Table 2 shows, for each study group, the serum Fis1, parkin, Mfn2 and PGC-1α levels; Fis1/parkin, Fis1/Mfn2, and Fis1/ PGC-1α ratios; and the PCT level. Figure 2 presents the Fis1/parkin, Fis1/Mfn2, Fis1/ PGC-1α ratios and serum PGC-1α, Fis1, Mfn2, parkin levels for each subgroup (infection, sepsis nonshock and septic shock groups). Serum PCT, Fis1, parkin, Mfn2, and PGC-1α levels, as well as the Fis1/parkin, Fis1/Mfn2, and Fis1/ PGC-1α ratios, at ICU admission differed significantly among the groups. Relative to the control group, the sepsis group had significantly higher serum mediator levels and ratios (*p* < 0.001). Additionally, the Fis1/parkin, Fis1/Mfn2, Fis1/ PGC-1α ratios and serum PGC-1α, Fis1, Mfn2, parkin levels were markedly different among the sepsis non-shock, septic shock, and control groups (*p* < 0.001). Moreover, for patients with sepsis, we compared the ratios and serum levels between survivors and non-survivors. We found that all three ratios were significantly lower in survivors compared to non-survivors (Figure 3). Survivors had significantly higher serum parkin, PGC-1α levels and lower Fis1 level.

**Table 2:** Mediators of the study population.

	**Infection**	**Sepsis**	***P*-value**
Number of subjects	24	133	
**Mediator levels**			
PCT (ng/mL)	0.82 (0.56–2.20)	7.00 (2.10–25.00)	<0.001
Fis1 (pg/mL)	523.10 (427.57–609.59)	761.27 (642.26–837.11)	<0.001
Parkin (pg/mL)	249.60 (232.89–268.21)	181.44 (166.49–201.67)	<0.001
Mfn2 (pg/mL)	226.85 (205.96–240.59)	131.37 (113.54–161.88)	<0.001
PGC-1α (pg/mL)	386.66 (356.09–408.83)	283.20 (252.35–324.50)	<0.001
Fis1/Parkin ratio	1.98 (1.75–2.49)	4.28 (3.22–5.10)	<0.001
Fis1/Mfn2 ratio	2.37 (2.00–2.69)	5.90 (4.04–7.32)	<0.001
Fis1/PGC-1α ratio	1.36 (1.14–1.52)	2.69 (2.05–3.17)	<0.001

*Date were presented by mean ± standard deviation, n (%) or median (interquartile range)*.

*PCT, procalcitonin; Fis1, fission 1; Mnf2, mitofusin2; PGC-1α: peroxisome proliferator-activated receptor γ coactivator 1α*.

### Value of Serum Levels and Ratios for Predicting 28-Day Mortality

**Figure 4:**
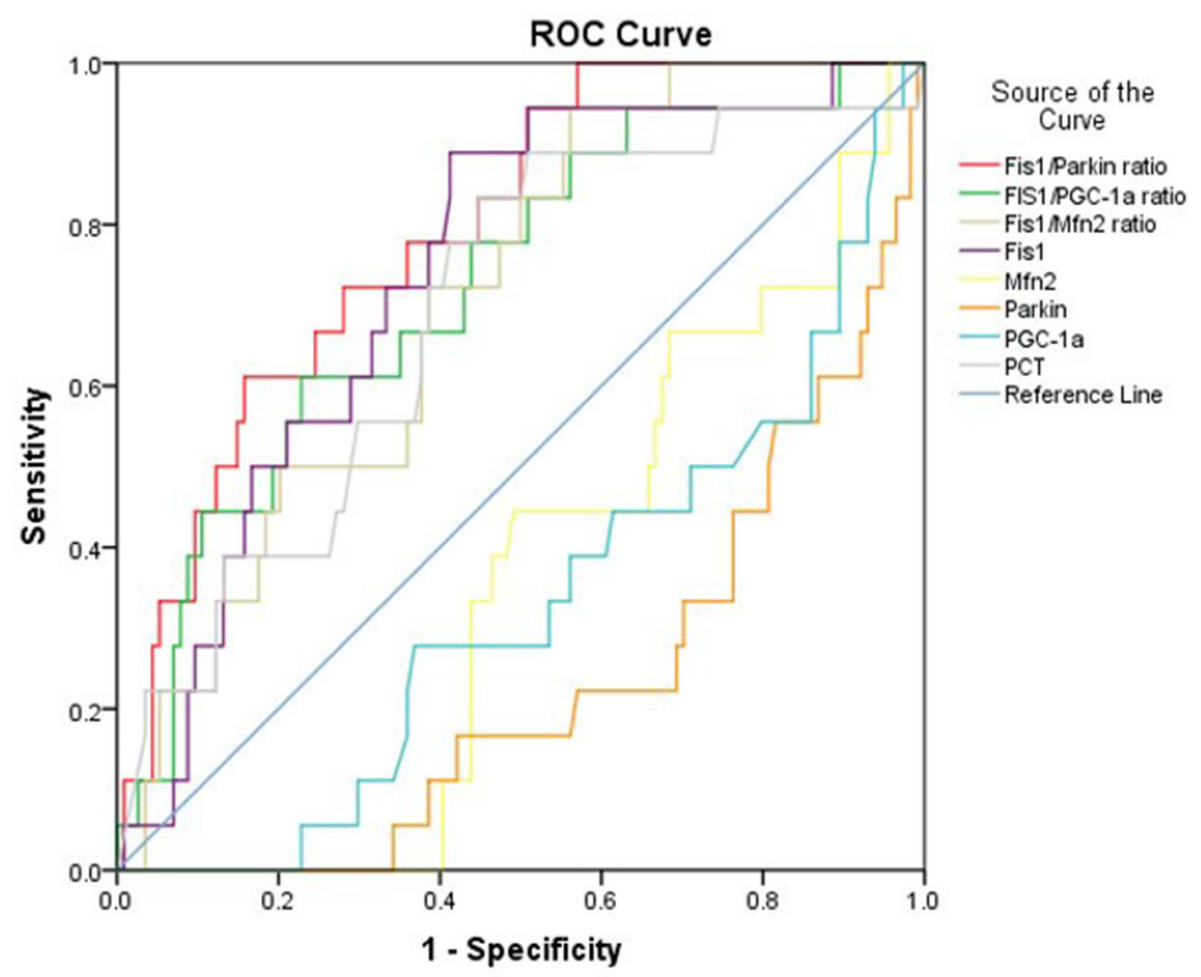
ROC curves for predicting 28-day mortality in patients with sepsis.

Considering that the ratios differed significantly between survivors and non-survivors, we calculated the AUCs of the mediator levels and ratios as predictors of 28-day mortality using the ROC curve. The results are presented in Table 3. As shown, the Fis1/parkin ratio was a better predictor than serum PCT, Fis1, parkin, Mfn2, and PGC-1α levels or Fis1/Mfn2 and Fis1/ PGC-1α ratios (Figure 4). For serum Fis1/parkin ratio at ICU admission, the AUC to predict 28-day mortality was 0.792 (95% CI, 0.695–0.890, *P* < 0.001), and the optimal cut-off value was 4.0 (sensitivity 94.4%, specificity 49.6%).

**Table 3:** AUCs for predicting 28-day mortality in patients with sepsis.

	**Variable**	**AUC**	**Standard error**	***P* value**	**95% CI**	
					**Lower limit**	**Upper limit**
28-day	Fis1/Parkin ratio	0.792	0.050	0.000	0.695	0.890
mortality	Fis1/Mfn2 ratio	0.708	0.056	0.005	0.598	0.817
	Fis1/PGC-1α ratio	0.725	0.063	0.002	0.601	0.848
	Fis1	0.740	0.056	0.001	0.630	0.849
	Parkin	0.231	0.055	0.000	0.124	0.338
	Mfn2	0.356	0.058	0.050	0.242	0.470
	PGC-1α	0.329	0.065	0.020	0.201	0.457
	PCT	0.692	0.066	0.009	0.563	0.821

### Fis1/parkin Ratio as an Independent Predictor of 28-Day Mortality

Next, the SOFA score, APACHE II score, and Fis1/parkin, Fis1/Mfn2, and Fis1/PGC-1α ratios were included in a multivariate logistic regression model to determine the independent predictors of 28-day mortality (Table 4). A high Fis1/parkin ratio (β = 1.347, odds ratio [OR] = 3.845, *p* = 0.021) was a significant independent risk factor for 28-day mortality in septic patients.

**Table 4:** Multivariate logistic regression analysis of factors predicting 28-day mortality in sepsis patients.

	**β**	**SE**	**Wald**	**OR**	***P*-value**
Fis1/Parkin ratio	1.347	0.582	5.363	3.845	0.021
Fis1/Mfn2 ratio	−0.738	0.364	4.114	0.478	0.043
Fis1/PGC-1α ratio	0.407	0.564	0.522	1.503	0.470
SOFA score	0.164	0.085	3.708	1.178	0.054
APACHE II score	0.115	0.040	8.129	1.122	0.004

### Survival

**Figure 5:**
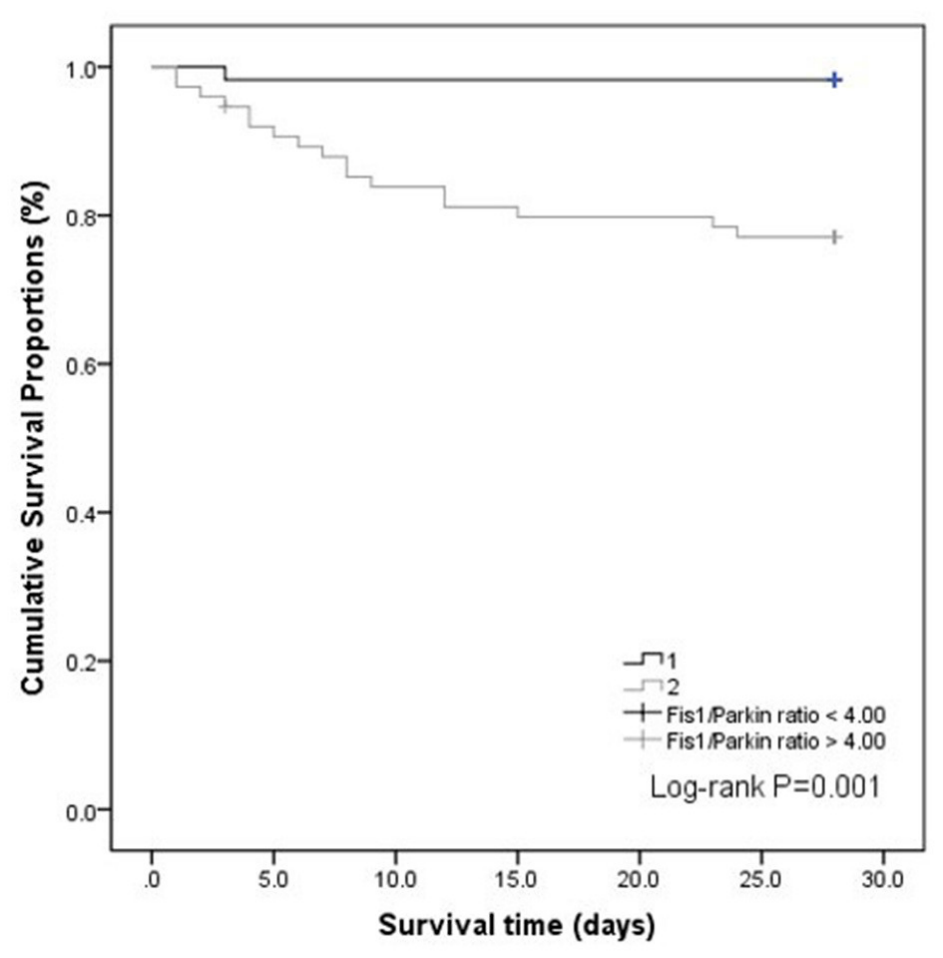
Kaplan–Meier survival curves of the Fis1/parkin ratio for all patients with sepsis.

We also performed a Kaplan–Meier survival analysis using the Fis1/parkin ratio cut-off (4.00) in septic patients (Figure 5). The results indicated that septic patients with a Fis1/parkin ratio above the cut-off value had a significantly lower survival rate (*p* = 0.001).

## Discussion

There is an urgent need for biomarkers to improve early diagnosis and risk stratification for patients with sepsis. In our study, patients with sepsis presented with significantly decreased serum levels of parkin, Mfn2, and PGC-1α and significantly increased serum Fis1 level and Fis1/parkin, Fis1/Mfn2, Fis1/PGC-1α ratios. Notably, higher ratios were associated with increased severity of disease. Relative to ICU patients with other infections, septic patients showed remarkably elevated ratios, with septic shock patients having the highest ratios. It has been proved in the clinical and experimental studies, that the persistence of low mitochondrial mitophagy, biogenesis, fusion levels and a high level of fission during sepsis suggested a poor outcome (3, 6, 14, 22), which agrees with the changes observed in our study.

Our findings support that high ratios at ICU admission can identify the severity of sepsis. In addition, septic patients with poor outcomes (non-survivors) had higher Fis1/parkin, Fis1/Mfn2, and Fis1/PGC-1α ratios. The ratios were greater than the absolute Fis1, parkin, Mfn2, and PGC-1α levels for prognostic purposes and had significant prognostic value for 28-day mortality. The Fis1/parkin ratio performed best among all of the ratios, as reflected by the AUC. Additionally, Fis1/parkin ratio was proved to be an independent risk predictor for patients with sepsis. Septic patients with a higher Fis1/parkin ratio (>4.0) had a significantly lower survival rate. Therefore, we chosen Fis1/parkin ratio to represent the mitochondrial homeostasis, indicating the balance between risk factor and protective factor.

Mitochondria are highly dynamic organelles and frequently undergo fission and fusion to modulate mitochondrial morphology, number, and size. Fission (in which mitochondria divide) is essential for cell growth and division, as it promotes adequate numbers of mitochondria. Fission may also occur when there is significant mitochondrial damage, as fission will allow the cell to segregate the damaged portion (23). During sepsis, mitochondrial dysfunction results in activation of mitochondrial fission and inactivation of mitochondrial fusion, which each or both promote dysfunctional mitochondrial fragmentation (13). It remains uncertain whether the elevation of fission is a physiological adaptation or a manifestation of decompensation under septic conditions (4), although persistent elevation of fission during sepsis is associated with the deterioration of sepsis both *in vivo* and *in vitro* (13). The pretreatment with fission inhibitor (mdivi-1) significantly attenuated mitochondrial dysfunction and apoptosis in septic animal model (3). In this profile, the overactivation of fission during sepsis may be a risk factor. Mitophagy refers to selective autophagy of mitochondria, and its main function is to recognize damaged mitochondria for degradation. In sepsis, mitochondrial dysfunction induces a loss of mitochondrial membrane potential that triggers mitophagy. Upregulation of mitophagy may improve organ function and reduce organ inflammation in response to lipopolysaccharide (LPS), whereas inhibition of mitophagy has been shown to increase mortality in septic mice (9, 22, 24). This phenomenon suggests a protective role of mitophagy during sepsis. Therefore, a higher Fis1/parkin ratio is expected to reflect a more severe imbalance of mitochondrial homeostasis. This would result in poorer outcomes for patients, which is consistent with our results. An elevation of Fis1/parkin ratio could be used to alert clinicians that mitochondrial dysfunction is existed, and clinical adjustments are needed to prevent the progression of sepsis. The reasons for the elevation of MQC-related biomarkers may be considered as: the persistent mitochondrial damage and the cell death (apoptosis, pyroptosis, or necroptosis) under septic exposure, which lead to continuous release of MQC-related proteins into circulation. In a clinical study in multiple sclerosis (MS) patients, the serum levels of parkin are remarkably augmented and associated with disease activity, indicating the use of parkin as biomarker of mitophagy (15). The researchers had previously proved that parkin levels were increased within the CNS and at the systemic level in patients with MS compared to other neurological disorders and healthy individuals (16). Though these studies were not in septic setting, but suggesting the possibility of use serum parkin level as mitophagy related biomarker in septic patients. There are also some studies investigated the fission protein (Fis1, Drp1) in peripheral blood lymphocytes (PBL) and also the gene and protein expression of mitochondrial fusion (Mfn2)/fission (Fis1)/biogenesis (PGC-1α) in peripheral blood mononuclear cells (PBMCs) as biomarker to reflect the MQC status (17–19). Immune cells may be the main source of MQC-related proteins release. Of course, further reevaluation of the patient and repeated exploration for a possible source are still needed, and our research is ongoing.

Given the crucial role of mitochondria in sepsis (25, 26), numerous studies have evaluated other mitochondrial function biomarkers, such as electron transport chain (ETC) enzyme activity in platelets or circulating mitochondrial DNA (mtDNA) in plasma or serum, to monitor mitochondrial function. However, ETC has not been found to be useful for evaluating the prognosis of sepsis (27, 28). Quite a few studies have investigated the prognostic value of mtDNA in septic patients (29–33). Most studies that performed AUC analysis found a statistically significant association between mtDNA levels and mortality. However, steps need to be taken to standardize how mtDNA is measured to facilitate large, prospective, multicenter trials to better assess the ability of mtDNA to predict outcomes. In a previous study, we evaluated the potential utility of serum uncoupling protein-2 (UCP2) level as a mitochondrial function biomarker in septic patients (34). However, it did not outperform the Fis1/parkin ratio (the AUC of serum UCP2 level for predicting 28-day mortality was 0.704, compared with 0.792 for the Fis1/parkin ratio). There are also studies investigated the mitochondria in circulating cells, such as respiratory chain biochemistry in platelets (35) or the complex activity in platelets (28) or mitochondrial bioenergetic reserve in PBMCs (36, 37), aiming to provide insight into sepsis-associated temporal changes and how these changes relate to recovery. However, they were all required further validation. At present, monitoring of mitochondrial function is still limited to experimental work.

In a word, use of the serum Fis1/parkin ratio has several advantages: (1) Serum Fis1 and parkin levels can be accurately determined with high reproducibility using the ELISA technique, relative to biomarkers measured by western blot (WB) or polymerase chain reaction (PCR). (2) Serum samples are easier to obtain than tissue/organ samples or blood mitochondria containing cells (i.e., immune cells). (3) The Fis1/parkin ratio is specifically related to sepsis pathophysiology rather than more general inflammatory reactions. The use of this biomarker may help transform our understanding of sepsis from a “physiological syndrome” to a “group of distinct biochemical disorders” and lead to advances in the search for adjunctive sepsis therapies (38).

### Limitations

Some limitations of this study merit consideration. First, an important limitation of the study is that the source of the proteins identified in the serum samples was not validated. Second, we did not compare the Fis1/parkin ratio with other classic biomarkers of mitochondrial function (i.e., mtDNA, complex of respiratory chain). Third, comparisons of Fis1 and parkin levels before and after treatment were not performed during patients' stays in the ICU. Fourth, complications of the patients' underlying diseases may have had an effect on the results. Lastly, this study was conducted at a single center with a relatively small sample size. Thus, larger studies at multiple centers are warranted to confirm our findings.

## Conclusions

This study aimed to assess the use of the serum Fis1/parkin ratio as a biomarker of prognosis in septic patients. We found that the serum Fis1/parkin ratio is an independent risk factor and a predictor of 28-day mortality in patients with sepsis. The higher the Fis1/parkin ratio is, the severer the disease is. These results suggest that this ratio is valuable for rapid risk stratification when patients are admitted to the ICU. Further study is needed to verify the potential utility and beneficial effects of this biomarker to answer specific clinical questions.

## Data Availability Statement

The raw data supporting the conclusions of this article will be made available by the authors, without undue reservation.

## Ethics Statement

The studies involving human participants were reviewed and approved by This study was approved by the Ethical Committee of PUMCH (approval number: JS-2421). The patients/participants provided their written informed consent to participate in this study.

## Author Contributions

WH conceived and designed the study, enrolled the patients, collected the blood samples, analyzed and interpreted data, performed the statistical analysis, and drafted the manuscript. XW conceived and designed the study, enrolled the patients, interpreted data and revised the manuscript. HZ enrolled the patients, analyzed data and revised the manuscript. GW enrolled the patients, collected blood samples, and analyzed data. DL conceived and designed the study, interpreted data and revised the manuscript. All authors read and approved the final manuscript.

## Conflict of Interest

The authors declare that the research was conducted in the absence of any commercial or financial relationships that could be construed as a potential conflict of interest.

## Abbreviations

MQC: mitochondrial quality control
PGC-1α: peroxisome proliferator-activated receptor γ coactivator 1α
Fis1: fission protein 1
Mfn2: mitofusin2
ICU: intensive care unit (ICU)
APACHE II score: Acute Physiology and Chronic Health Evaluation II score
CRRT: continuous renal replacement therapy
HR: heart rate
MAP: mean arterial blood pressure
MV: mechanical ventilation
NE: norepinephrine
SOFA: sequential organ failure assessment score
CVP: central venous pressure
Lac: lactic acid
PaO2: arterial partial pressure of oxygen
PI: perfusion index
Pv-aCO2: arteriovenous carbon dioxide partial pressure difference
ScvO2: central venous blood oxygen saturation
PCT: procalcitonin
cTnI: cardiac troponin I
NT-ProBNP: N-terminal pro-brain natriuretic peptide.
